# Using neuroimaging to investigate the impact of Mandolean® training in young people with obesity: a pilot randomised controlled trial

**DOI:** 10.1186/s12887-018-1342-1

**Published:** 2018-11-22

**Authors:** Elanor C. Hinton, Laura A. Birch, John Barton, Jeffrey M. P. Holly, Kalina M. Biernacka, Sam D. Leary, Aileen Wilson, Olivia S. Byrom, Julian P. Hamilton-Shield

**Affiliations:** 1 NIHR Bristol Biomedical Research Centre Nutrition Theme, University of Bristol, University Hospitals Bristol Education & Research Centre, Upper Maudlin Street, Bristol, BS2 8AE UK; 2 Clinical Research and Imaging Centre (CRICBristol), 60 St Michael’s Hill, Bristol, BS2 8DX UK; 3 Department of Paediatric Endocrinology and Diabetes, Bristol Royal Hospital for Children, Upper Maudlin Street, Bristol, UK; 4 School of Translational Health Sciences, IGFs and Metabolic Endocrinology, University of Bristol, Second Floor, Learning and Research, Southmead Hospital, Westbury-on-Trym, Bristol, BS10 5NB UK

**Keywords:** Eating rate, Satiety, fMRI, Adolescents, Obesity, Brain

## Abstract

**Background:**

Slowing eating rate using the Mandolean® previously helped obese adolescents to self-select smaller portion sizes, with no reduction in satiety, and enhanced ghrelin suppression. The objective of this pilot, randomised trial was to investigate the neural response to food cues following Mandolean® training using functional Magnetic Resonance Imaging (fMRI), and measures of ghrelin, PYY, glucose and self-reported appetite.

**Method:**

Twenty-four obese adolescents (11–18 years; BMI ≥ 95th centile) were randomised (but stratified by age and gender) to receive six-months of standard care in an obesity clinic, or standard care plus short-term Mandolean® training. Two fMRI sessions were conducted: at baseline and post-intervention. These sessions were structured as an oral glucose tolerance test, with food cue-reactivity fMRI, cannulation for blood samples, and appetite ratings taken at baseline, 30 (no fMRI), 60 and 90 min post-glucose. As this was a pilot trial, a conservative approach to the statistical analysis of the behavioural data used Cliff’s delta as a non-parametric measure of effect size between groups. fMRI data was analysed using non-parametric permutation analysis (RANDOMISE, FSL).

**Results:**

Following Mandolean® training: (i) relatively less activation was seen in brain regions associated with food cue reactivity after glucose consumption compared to standard care group; (ii) 22% reduction in self-selected portion size was found with no reduction in post-meal satiety. However, usage of the Mandolean® by the young people involved was variable and considerably less than planned at the outset (on average, 28 meals with the Mandolean® over six-months).

**Conclusion:**

This pilot trial provides preliminary evidence that Mandolean® training may be associated with changes in how food cues in the environment are processed, supporting previous studies showing a reduction in portion size with no reduction in satiety. In this regard, the study supports targeting eating behaviour in weight-management interventions in young people. However, given the variable usage of the Mandolean® during the trial, further work is required to design more engaging interventions reducing eating speed.

**Trial registration:**

ISRCTN, ISRCTN84202126, retrospectively registered 22/02/2018.

## Background

Newly reported global childhood obesity levels highlight the importance of focussing on young people (children and adolescents) in weight-management research [1]. Encouraging adaptive eating behaviour early may provide young people with additional skills to take into adulthood, over and above messages of improving diet and exercise. Indeed, evidence suggests that targeting eating behaviour may be an effective strategy [2, 3]; for example, slowing eating rate has been shown to reduce energy intake [4, 5]. Moreover, a trial of the Mandolean®, a computerised ‘meal’ weighing device that provides contemporaneous feedback and purposely trains the participant to eat more slowly over time, be mindful of developing fullness and reduce portion size, demonstrated a reduction in body mass index (BMI) in obese children when used in combination with a weight-management programme [6]. Mandolean® training in young people was associated with enhanced suppression of ghrelin and increased PYY post-meal [7], and smaller self-selected portion sizes with the same post-meal satiety than before training [6, 7].

Research is increasingly pointing to the utility of neuroimaging techniques, such as food cue-reactivity functional Magnetic Resonance Imaging (fMRI), to understand the mechanisms underlying changes following weight-management interventions [8–12]. FMRI food cue-reactivity has been conducted in the fasted state and following energy intake, e.g. through consumption of a standard meal or a meal based on individual energy requirements. Oral glucose tolerance tests (consumption of a fixed glucose load/kg) provide a controlled protocol with known physiological effects with which to measure the associated neural response to food cues following energy intake [13, 14]. Previous research has indicated brain regions involved in the response to food cues and consumption of glucose to include insula [15], hypothalamus [16], amygdala [17, 18], striatum [11, 19], orbitofrontal cortex (OFC) [17, 20], and the temporal occipital fusiform cortex (TOFC) [13, 14]. The neural mechanisms underlying the changes following Mandolean® training are yet unknown, leading to the current research question of how such a behavioural intervention to slow eating rate may affect the neural processing of food cues in the environment.

To address this question, a two-arm pilot randomised controlled trial was designed, with obese, adolescent patients randomised to receive either Mandolean® training plus six-months standard care in an obesity clinic, or six-months standard care. Baseline and post-intervention oral glucose tolerance tests were conducted, including measurements of food cue-reactivity fMRI, gastrointestinal hormones and self-reported appetite. The objectives of this pilot randomised controlled trial were two-fold: first, to assess the feasibility of conducting a larger-scale trial of the Mandolean® using changes in fMRI measures as one of the outcomes (in addition to BMI change). Feasibility outcomes were usage of the Mandolean® (number of meals consumed using the device), tolerance of the imaging protocol (drop-out rate) and blood sampling protocol (number of patients from whom samples were taken), and ability to measure imaging signal in the brain regions of interest. Secondary objectives were to provide preliminary data of the impact of Mandolean® training, which aims to slow eating rate and reduce portion size, on the neural response to food cues following glucose consumption in adolescents with obesity, measured using fMRI.

## Materials and methods

### Participants

Twenty-four adolescents (11–18 years; BMI ≥ 95th centile) were recruited from the Care of Childhood Obesity clinic at Bristol Royal Hospital for Children (Table 1). Exclusion criteria were as follows: diagnosed learning difficulties, visual or hearing difficulties, dysmorphic features suggestive of syndromic obesity such as Prader–Willi Syndrome; endocrine disorders; iatrogenic causes of obesity; MRI contraindications e.g. metal implants, pregnancy, history of neurological disease, traumatic brain injury, mental illness, claustrophobia, medications that may disrupt appetite, weight above 152 kg due to the limits of the scanner bed, and girth of more than 210 cm (to ensure fit inside the 70 cm diameter bore of the scanner); vegetarian or vegan (so that the images of food shown in the cue-reactivity task were not aversive to participants). Parents gave written informed consent for their child to participate, and participants gave assent. The study was approved by the Frenchay NHS Ethics Committee (13/SW/0076). The sample size of this feasibility study was determined through consideration of the number of potentially eligible participants attending the clinic during the study period and by consulting existing literature reporting pilot feasibility trials such as this (e.g. [11]).

**Table 1:** Participant details

Measures (median (IQR))	Mandolean+ group			Standard care group			Cliff’s delta (C.I.)
	Baseline	Post-Intervention	Mean % difference (C.I.)^a^	Baseline	Post-Intervention	Mean % difference (C.I.)^a^	(Post-Intervention)
N	10	10	10	9	9	9	
Age (years)	13.00 (5.00)	13.00 (4.50)	–	13.00 (3.00)	14.00 (4.00)	–	0.08 (−0.50, 0.48)
Gender (M/F)	4/6	4/6	–	3/6	3/6	–	–
BMI SDS	3.31 (0.92)	3.38 (1.07)	−1.16 (−4.17, 1.85)	3.25 (0.51)	3.15 (0.44)	−2.37 (−5.50, 0.76)	0.2 (−0.36, 0.65)

^a^ Mean % difference within groups: ((Post-Intervention value - Baseline value)/Baseline value)*100

### Study design and measures

Participants were randomised based on age and gender to receive 6 months of standard care (standard care group), or standard care plus Mandolean® training (Mandolean+ group). Standard care in the obesity clinic typically comprised two clinic appointments with a clinician, dietitian and exercise specialist over the six-month period. Participants in the Mandolean® + group received additional training on how to use the device (described elsewhere [6]). In brief, participants were asked to use the device for their main meal of the day as many times as possible in the six-month period. Participants and their parents were given advice regarding the types of suitable meals (i.e. those eaten with cutlery) and meals to avoid when using the Mandolean® (e.g. burgers/sandwiches as the food is lifted off the plate for each mouthful, reducing utility). Participants placed their empty plate on the Mandolean weighing scale at the start of the meal. The device then prompted the user to add food to an individually pre-programmed quantity and recorded this portion size. The Mandolean then recorded how fast the food was removed from the plate while the meal was being eaten. The computer audibly prompted the user to slow down if the food was removed faster than a pre-specified eating rate in order to ‘train’ the individual to reduce their speed of eating. The computer also prompted the subject to rate level of satiety regularly during the meal (a form of mindfulness of eating). More information about the validation of the device can be found here [21] (https://mando.se/en/mandometer-method/).

At baseline and post-intervention, participants underwent two neuroimaging sessions at Clinical Research and Imaging Centre (CRICBristol). Sessions involved an oral glucose tolerance test (75 g glucose in 436 ml drink), in which the blood oxygen level dependent (BOLD) response during a food cue-reactivity task, appetite ratings, glucose, ghrelin and PYY levels were measured at baseline and 30- (no BOLD), 60- and 90-min post consumption of the glucose drink. Self-reported appetite (How hungry/full/thirsty do you feel right now?) was assessed using 7-point Likert scales, with the end points ‘Not at all’ and ‘Extremely’. Measurements of height and weight were taken to calculate BMI SDS at each session.

Using an event-related design, the food cue reactivity task presented 90 food images and 45 non-food images (e.g. household objects) for 3 s each; with variable length null events to provide jitter between images. Images were slightly offset from the centre of the screen and participants indicated whether the image was on the left or right of the screen using a button box inside the scanner. After every 20 food pictures, a feedback trial was presented to participants based on their responses to the preceding images, with one of the following messages: “Well done! Keep going!” (13 or more correct responses); “Well done! Please try to press the correct button for each picture” (between 7 and 12 correct responses); “Please pay close attention to the pictures and try to press the correct button” (less than 7/20 correct responses). Food images included sweet and savoury foods that varied in energy content and incentive value. Stimuli had previously been independently rated [22], with food and non-food images matched as closely as possible for size, colours and visual complexity, as per another previous study [23]. All food images were rated on liking and familiarity by participants prior to the scan, using an online survey designed for the study. A differential number of food and non-food images were included in the analysis to include 45 food images each of high and low incentive value to the participant (as per (18)).

Following each session, participants in both groups were asked to consume three meals using the Mandolean® at home. For each meal, the Mandolean® recorded the self-selected portion size (g), amount consumed (g), duration of the meal (minutes), and self-reported satiety at the start of the meal. On a separate sheet, participants recorded what foods they had consumed, and their self-reported satiety at the end of the meal. N.B. For these test meals, the device did not provide a pre-programmed portion size guide or provide feedback on eating rate or satiety during the meal.

### Blood sample preparation and analysis

Blood samples were collected into aprotinin containing EDTA tubes, inverted and centrifuged in 4 °C at 2500 rpm for 15 min. 1 N hydrochloric acid (HCl) and phenylmethylsulfonyl fluoride (PMSF) were added as preservatives. Plasma samples were kept in − 80 °C until assayed. Total active ghrelin levels were measured by radioimmunoassay (RIA) according to protocol recommendations using a standard curve of known concentration of purified 125I-labeled ghrelin peptide (GHRA-88HK; EMD Millipore Corporation). No plasma dilution was applied when measuring ghrelin levels. The coefficient of variance (CV) for intra-assay variability was 5.2% and the CV for inter-assay variability was 5.5%. Total PYY levels were measured by radioimmunoassay (RIA) according to protocol recommendations using a standard curve of known concentration of purified 125I-labeled PYY peptide (PYYT-66HK; EMD Millipore Corporation). No plasma dilution was applied when measuring PYY levels. The coefficient of variance (CV) for intra-assay variability was 3.3% and inter-assay variability was 7.6%. Plasma glucose levels were obtained using Glucose Assay Kit II (Abnova Corporation, Taiwan). Plasma samples were kept in − 80 °C until assayed. Plasma samples were diluted 4 times for the best standard curve fit. The coefficient of variance (CV) for intra-assay variability for was 4.3% and the CV for inter-assay variability was 5.2%.

### Statistical analysis of behavioural data

As the data were non-normally distributed, a non-parametric measure of effect size is reported, along with 95% confidence intervals for the estimate (Cliff’s delta, d [24]), calculated using a new Excel macro [25]. Spearman’s Rho is reported for the correlation between Mandolean® usage and (i) % signal change in striatum and TOFC post-intervention and (ii) BMI change. Statistical tests were not performed on this data due to a lack of power in the pilot trial.

### fMRI data acquisition and analysis

Neuroimaging took place at CRICBristol on a Siemens 3 T Magnetom Skyra MRI scanner using a 32-channel head coil. Functional MR images were acquired in one run using a BOLD EPI sequence. Details of parameters are as follows: TR = 2520 ms; TE = 30 ms; flip angle = 90°; FOV = 192; no. of slices = 45 with 25% gap, interleaved; voxel size = 3 × 3 × 3 mm; phase encoding = A> > P; phase oversampling = 0%; GRAPPA = ON with acceleration factor PE = 2; bandwidth = 2368 Hz/Px; no. of volumes = 260; duration = 11:03 min. High resolution structural scan was acquired (MPRAGE), with the following parameters: TR = 2300 ms; TE = 2.98 ms; flip angle = 9°; FOV = 256; no. of slices = 192 (3D volume scan); voxel size = 1 × 1 × 1.1 mm; inversion time = 900 ms; phase oversampling = 0%; GRAPPA = ON with acceleration factor PE = 2; bandwidth = 240 Hz/Px; no. of volumes = Single shot; duration = 5:12 min.

Pre-processing and first level analysis of functional images was performed using FMRIBs Expert Analysis Tool (FEAT) [26]. Standard pre-processing steps were followed: motion correction using MCFLIRT [27], non-brain removal using BET [28], spatial smoothing using a Gaussian kernel of FWHM5 mm, mean-based intensity normalisation of all volumes, high-pass temporal filtering. In addition, the tool ICA-AROMA was utilised to remove further motion-related artefact from the data [29]. Registration was optimised by using high-resolution field-maps to correct for distortions in the EPI data [30]. Registration to high resolution and standard images was carried out using FMRIB’s Linear Image Registration Tool (FLIRT [31]), then registration from high resolution structural to standard space was refined using FNIRT nonlinear registration [32, 33]. At the first level, time-series statistical analysis was carried out using FMRIBs Improved Linear Model (FILM) with local autocorrelation correction (prewhitening) [34] on the each separate scan taken at baseline, at 60 min post glucose, and at 90 min post glucose. Z statistic images were thresholded using clusters determined by Z > 2.3 and a (corrected) cluster significance threshold of *P* = 0.05 [35]. Explanatory variables were added to the general linear model for each type of food picture (high incentive food, low incentive food, non-food), as well as the feedback trials (not analysed further). Contrasts were defined to examine the response to each image type, the comparison between high and low incentive foods, and most importantly, the response to food cues (high and low incentive together) minus the response to non-food cues. These contrast of parameter estimates (COPEs) were subsequently used to perform second-level group analyses. Contrasts of high and low incentive value did not produce any significant differences, therefore the group analysis presented below focusses on the contrast between food and non-food images.

Group-level statistical analysis was conducted with a masked approach using RANDOMISE, FSL’s tool for nonparametric permutation inference on neuroimaging data [36]. A priori regions of interest were selected as masks based on previous literature (see introduction). Bilateral ROIs were created by thresholding masks from the Harvard-Oxford Cortical and Subcortical structural atlases in FSLview, except the hypothalamus mask that was drawn by hand using the Atlas of the Human Brain [37] as a guide. The RANDOMISE analysis used the food-non-food COPE only taken from the first level analyses and transformed into standard space (as described above). First, the response at baseline was subtracted from (i) the response at 60 min post glucose, and (ii) the response at 90 min post glucose. These difference images were fed into the RANDOMISE analysis to conduct unpaired t-tests between the Mandolean® + and standard care groups, using the TFCE (Threshold-Free Cluster Enhancement) cluster-based analysis option, and a FWE-corrected *p* values thresholded at *p* < 0.05. Cluster and peak data was extracted by masking the raw stats image with the significant voxels from the corrected stats image, then extracting the cluster information using the ‘cluster’ command (as recommended on FSL Randomise User guide). The closest to estimates of effect size in fMRI data is to extract the percentage BOLD signal change in the regions of interest and plot the values for each group. As this was a pilot study with a small sample size, no correction for multiple comparisons has been applied (to account for the number of tests done over masks), so the results of these analyses should be considered preliminary. (NB. Analysis of the impact of glucose on neural food cue-reactivity comparing participants of a healthy weight and obesity is in preparation).

## Results

Only those participants with data from both the baseline and post-intervention session were included in the analyses (except for the Mandolean data in Table 3). The samples included at each time point (baseline and post-intervention) are described in Table 1. Five participants disengaged from the study following the first imaging session (four from Mandolean® + and one from standard care group) for various reasons (illness, relocation, insufficient time for intervention, lost to follow up).

### Feasibility outcomes

Tolerance to the imaging protocol was measured by drop-out rates from the study. Twenty-four participants began the first imaging session. As described above, three participants dropped out from the study due to reasons other than the imaging protocol. Two participants were lost to follow up, both of whom struggled with the imaging protocol during the first session: one needed her mother to be in the magnet room with her and found keeping still for the MRI uncomfortable; the other refused to return to the scanner for the second scan during the first session. Overall, a high percentage (79%) completed both imaging sessions.

Adherence to the blood sampling protocol was more challenging. Cannulation was difficult to achieve in this patient group. 13/24 (54.2%) were cannulated in the baseline session, of whom eight were cannulated in the post-intervention session. Therefore, blood samples from the post-intervention session were analysed for eight participants only (four in each group; 33.3%).

Usage of the Mandolean® was measured by the number of meals the device was used during the intervention period. A median of 28.0 (IQR = 54.5) meals with usable data over six-months was found, but with a large range: one participant only recorded five meals with the device, whereas another recorded 80 meals with the device. Due to problems with the device, data was not saved for all meals; a problem that affected 15% meals during the intervention for the Mandolean+ group. This also affected whether there was saved test meal data for participants at baseline and/or post-intervention: 6/19 participants (32%) completed test meals but the data was not recorded. A further 3/19 participants (16%) did not attempt the post-intervention test meals.

Ability to measure imaging signal in the brain regions of interest was investigated through examination of the first level maps for each participant. These showed that signal change was observed in the regions of interest in the brain. There was some signal loss in the OFC (an area known to be susceptible to artefact due to proximity to air-filled sinuses), but a BOLD response was still seen in this key area. Field-maps were incorporated into the processing pipeline such that the data in this and other regions was corrected for distortions in the magnetic field.

### Preliminary results from post-intervention session

**Fig. 1:**
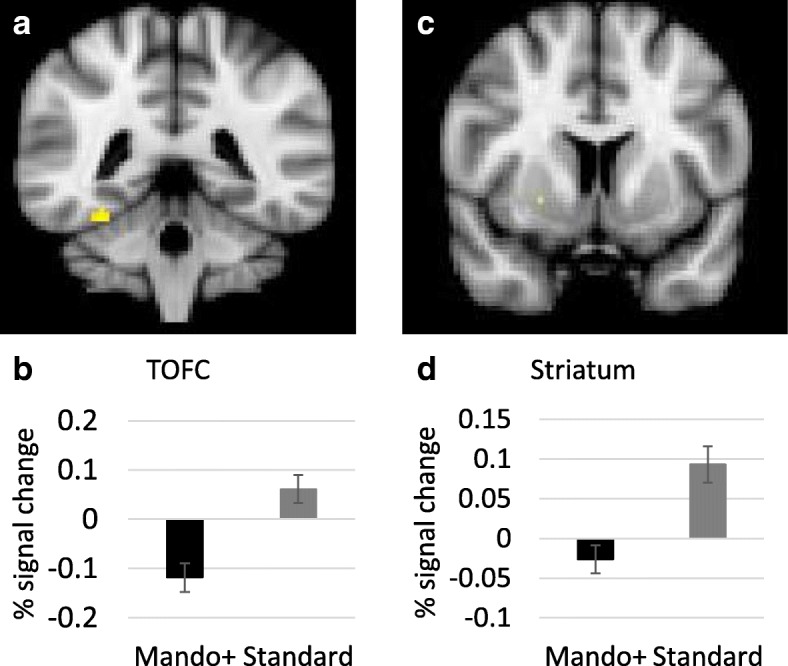
Clusters of reduced activation in the Mandolean® + group compared to the standard care group for the contrast between 60 min post-glucose and baseline in the Post-intervention session. **a** TOFC *t* = 3.88, x = 32, y = − 42, z = − 22, cluster size = 16 voxels); **b** Percentage signal change in the TOFC; **c** Putamen *t* = 4.29, x = 24, y = 24, z = − 4, cluster size = 4 voxels; **d** Percentage signal change in the putamen

The BOLD response to glucose (controlling for fasting response) during food cue-reactivity was compared between the Mandolean® + and standard care groups at baseline and post-intervention separately. No group differences were found during the baseline scan at 60- or 90-min post glucose, as expected. Post-intervention, signal change in the TOFC and a region of the striatum (putamen) 60 min post-glucose relative to fasting between intervention groups is shown in Fig. 1. Both these regions show greater reactivity to food cues 60 min post-glucose in the standard care group compared to the Mandolean® + group. No between-group differences at 60 min post glucose were found in other masks (insula, hypothalamus, amygdala and OFC). Activity in the putamen remained different between groups at 90 min post-glucose, with a cluster of differential activation in the putamen (*t* = 3.63, MNI brain co-ordinates: x = 24, y = 10, z = − 2, cluster size = 24 voxels). No between-group differences at 90 min post glucose were found in other masks (insula, hypothalamus, amygdala, OFC and TOFC).

During the post-intervention session, a greater change in fullness at 60 and at 90 min post-glucose from baseline in the Mandolean® + group compared to the standard care group was found, with smaller effect sizes for a difference in hunger and thirst (Table 2). Preliminary evidence for ghrelin suppression at 60 and at 90 min post-glucose from baseline in the Mandolean® + group compared to the standard care group was found (Table 2).

**Table 2:** OGTT variables

Measures (median (IQR))	Mandolean+ group			Standard care group			Cliff’s delta (C.I.)
	Baseline	Post-Intervention	Mean % difference (C.I.)^a^	Baseline	Post-Intervention	Mean % difference (C.I.)^a^	(Post-Intervention)
N with VAS ratings	10	10	10	9	9	9	
Fullness rating (0–7 Likert scale)							
Fasting	2.00 (2.00)	3.50 (1.25)	125.93 (−20.02, 271.87)	2.00 (1.50)	3.00 (1.00)	111.11 (32.40, 189.82)	0.21 (− 0.28, 0.62)
Post glucose load: 30 mins.	2.00 (3.00)	4.50 (2.50)	^b^-70.00 (−173.89, 33.89)	4.00 (2.50)	3.00 (1.00)	^b^-96.25 (− 146.22, − 46.28)	^b^0.27 (−0.24, 0.66)
60 min	1.50 (2.25)	4.00 (1.00)	^b^ − 100.00 (− 248.41, 48.41)	2.00 (1.50)	3.00 (2.00)	^b^ − 84.00 (− 189.94, 21.94)	^b^0.46 (− 0.08, 0.79)
90 min	1.00 (2.00)	5.00 (1.25)	^b^ − 112.50 (− 341.02, 116.02)	2.00 (3.50)	4.00 (0.50)	^b^ − 33.33 (− 119.02, 52.35)	^b^0.52 (0.02, 0.82)
Hunger rating (0–7 Likert scale)							
Fasting	5.00 (3.50)	1.00 (0.50)	−54.71 (−80.57, − 28.85)	4.00 (1.50)	2.00 (2.00)	−32.33 (− 66.60, 2.16)	0.46 (− 0.78, 0.05)
Post glucose load: 30 mins.	4.00 (1.50)	1.00 (1.00)	^b^ − 106.25 (− 121.03, − 91.47)	3.00 (2.50)	1.00 (1.00)	^b^ 45.24 (− 153.10, 243.58)	^b^0.26 (− 0.30, 0.69)
60 min	4.50 (2.75)	1.00 (0.25)	^b^ − 100.00 (− 153.40, − 46.60)	3.00 (2.00)	1.00 (0.50)	^b^ − 58.33 (− 165.44, 4.77)	^b^0.23 (− 0.33, 0.67)
90 min	4.00 (3.00)	1.00 (1.00)	^b^ − 107.14 (− 148.75, − 65.54)	4.00 (2.00)	1.00 (1.00)	^b^ − 71.43 (− 184.25, 41.39)	^b^0.31 (− 0.26, 0.71)
Thirst rating (0–7 Likert scale)							
Fasting	3.00 (1.50)	3.00 (2.00)	− 7.59 (− 48.69, 33.51)	4.00 (2.00)	2.00 (1.50)	−21.85 (−62.00, 18.30)	0.31 (− 0.23, 0.70)
Post glucose load: 30 mins.	2.00 (1.00)	2.00 (3.00)	^b^ − 63.33 (− 114.86, − 11.80)	3.00 (3.00)	4.00 (3.00)	^b^ − 17.86 (−155.76, 120.04)	^b^0.58 (− 0.85, − 0.07)
60 min	3.00 (1.00)	2.50 (2.25)	^b^ − 100.00 (− 252.07, 52.07)	2.00 (2.00)	3.00 (1.50)	^b^ − 140.48 (− 217.99, − 62.97)	^b^0.26 (− 0.65, 0.25)
90 min	3.00 (1.50)	2.00 (2.25)	^b^ − 33.33 (− 176.76, 110.09)	4.00 (2.00)	2.00 (1.50)	^b^ − 66.67 (− 121.02, − 12.31)	^b^0.39 (− 0.77, 0.19)
N with blood plasma data	5	4	4	5	4	4	
Ghrelin (pg/ml)							
Fasting	9.80 (8.25)	14.00 (9.90)	69.24 (− 38.24, 176.72)	14.30 (15.50)	12.40 (7.50)	10.49 (−99.79, 120.77)	0.63 (− 0.38, 0.95)
Post glucose load: 30 mins.	8.75 (6.58)	14.40 (5.50)	− 105.56 (− 271.44, 60.31)	10.60 (15.30)	13.70 (15.00)	94.21 (− 130.27, 318.69)	^c^0.13 (− 0.67, 0.78)
60 min	9.30 (8.20)	10.50 (4.20)	−13.42 (− 220.46, 193.61)	6.90 (6.80)	13.25 (7.80)	−78.25 (− 200.61, 44.11)	^c^0.75 (− 0.97, 0.21)
90 min	7.60 (9.10)	10.05 (6.60)	−7.08 (− 167.01, 152.87)	13.30 (17.95)	10.95 (10.80)	−58.32 (− 237.17, 120.52)	^c^0.75 (− 0.97, 0.21)
PYY (pg/ml)							
Fasting	79.30 (40.75)	79.50 (38.60)	−2.29 (−19.81, 15.23)	63.40 (50.55)	68.75 (73.3)	4.75 (−21.19, 30.70)	0.25 (− 0.61, 0.84)
Post glucose load: 30 mins.	81.45 (45.33)	83.65 (30.90)	− 174.86 (− 511.86, 162.15)	76.60 (15.95)	82.50 (35.00)	−10.98 (− 48.89, 26.93)	^c^0.13 (− 0.80, 0.69)
60 min	58.20 (28.90)	67.90 (29.70)	−32.74 (− 56.14, − 9.34)	63.20 (23.15)	60.10 (32.40)	−30.02 (− 264.99, 204.95)	^c^0.25 (− 0.61, 0.84)
90 min	55.00 (21.75)	62.70 (27.20)	−30.77 (− 79.01, 17.46)	59.50 (39.20)	83.45 (48.00)	745.41 (− 1610.16, 3100.98)	^c^0.50 (− 0.92, 0.43)
Glucose							
Fasting	6.00 (1.25)	6.4 (0.5)	1.22 (− 21.47, 23.92)	6.24 (0.25)	6.35 (1.48)	1.97 (−16.98, 20.91)	0.06 (− 0.70, 0.76)
Post glucose load: 30 mins.	10.00 (3.32)	9.55 (2.35)	7.46 (− 64.99, 79.90)	10.40 (4.95)	9.05 (3.52)	−87.53 (− 234.61, 59.54)	^c^0.51 (− 0.43, 0.92)
60 min	10.30 (5.20)	8.45 (1.60)	19.20 (− 200.77, 239.17)	7.30 (2.30)	7.75 (2.03)	−30.16 (− 283.69, 223.36)	^c^0.94 (0.35, 1.00)
90 min	8.40 (0.95)	8.01 (2.45)	−11.83 (− 118.84, 95.18)	8.20 (2.35)	7.3 (2.25)	−104.87 (− 367.08, 157.33)	^c^0.38 (− 0.52, 0.88)

^a^Mean % difference within groups: ((Post-Intervention value - Baseline value)/Baseline value)*100

^b^calculated on change from baseline scores

^c^calculated on % change from baseline scores

There was limited difference in BMI standard deviation score post-intervention between groups (Table 1), and within groups from baseline to post-intervention. However, 60% of the Mandolean® + group and 78% of the standard care group reduced their BMI during the intervention. There was only a 6 g difference in food intake in the post-intervention test meals between groups (Table 3). However, a 22% reduction in consumed portion size was identified in the Mandolean® + group (Table 3).

**Table 3:** Mandolean test meal variables

Measures (median (IQR))	Mandolean+ group			Standard care group			Cliff’s delta (C.I.)
	Baseline	Post-Intervention	Mean % difference (C.I.)^b^	Baseline	Post-Intervention	Mean % difference (C.I.)^b^	(Post-Intervention)
N with test meal data	7	4	2	9	7	7	
Meal Duration (min)	10.17 (6.17)	6.86 (7.55)	−3.46 (− 20.24, 13.33)	6.30 (1.36)	6.33 (1.84)	4.00 (− 15.68, 23.68)	0.57 (− 0.23, 0.91)
Portion weight (g)	473.00 (256.67)	294.00 (336.84)	−22.50 (−104.21, 59.22)	302.00 (58.65)	304.33 (137.59)	−12.02 (− 33.28, 9.23)	0 (−0.69, 0.69)
Meal portion consumed (g)	342.00 (159.00)	250.67 (309.88)	−14.40 (− 155.87, 127.07)	283.00 (71.53)	260.67 (163.67)	−13.61 (− 33.58, 6.35)	0.07 (−0.72, 0.65)
Eating rate (g/min)	33.81 (16.88)	34.10 (15.81)	−11.47 (− 142.58, 119.65)	48.10 (9.16)	41.23 (14.05)	−15.20 (−39.08, 8.68)	0.64 (−0.93, 0.12)
Premeal satiety (VAS^a^)	22.67 (13.84)	18.92 (16.55)	−70.51 (− 191.28, 50.27)	39.67 (34.79)	26.84 (28.42)	−20.84 (−64.74, 23.06)	0.75 (−0.97, 0.13)
Postmeal satiety (VAS^a^)	58.67 (33.75)	51.09 (52.29)	−14.06 (−40.24, 12.12)	47.00 (52.02)	56.67 (14.00)	130.22 (− 144.10, 404.54)	0 (−0.69, 0.69)

^a^ where 0 is not at all full, and 100 is extremely full)

^b^ Mean % difference within groups: ((Post-Intervention value - Baseline value)/Baseline value)*100

Finally, for the Mandolean® + group only, the relationships between Mandolean® usage and (i) the signal change in the two brain regions that showed differential response during the post-intervention scan, and (ii) BMI change, were investigated. A negative correlation was found between the number of meals eaten with Mandolean® and (i) signal change 60 min post-glucose compared to baseline in the TOFC (*r* = − 0.72) and striatum (*r* = − 0.29), and (ii) with BMISDS change (*r* = − 0.37). It appears that the more meals eaten using Mandolean®, the less reactivity (signal change) to food cues post glucose consumption is found, and a slightly greater reduction in BMI SDS.

## Discussion

We present preliminary evidence of a reduction in the neural response to food cues following glucose consumption in young people with obesity after Mandolean® training to slow eating rate. Reduced reactivity to food cues in the TOFC, part of the visual attention stream, in the Mandolean® + group may represent attenuated visual attention to food cues [8, 13]; an effect that may be mediated by insulin (e.g. [14]). Indeed, greater insulin levels have been associated with reduced neural food-cue reactivity in several studies [38, 39], leading to the speculation that insulin levels may be a putative physiological mechanism by which slowing eating rate impacts on brain activity and eating behaviour. Due to problems with cannulation however, it was not possible to measure insulin in the current study, but future work will incorporate additional physiological measurements.

Reduced reactivity post-glucose in the putamen is in keeping with previous research [14], and may suggest that responses to the rewarding food has changed for those in the Mandolean® + group, compared to those in the standard care group [40]. Indeed, a similar reduction in striatal response to high calorie food cues post behavioural intervention was found by Deckersbach et al. [11]. Neural reactivity to food cues (nucleus accumbens, also in reward pathway) has previously been shown to predict subsequent food intake [23]; therefore it is possible that, with less reactivity to food cues following energy intake, the Mandolean® + group may have less motivation to seek out and eat more food. Indeed, Mandolean® training was associated with a 22% reduction in portion size with no reduction in post-meal satiety. Strengthening this result is the link between the intervention and the BOLD response; specifically, the greater use of the Mandolean® saw less reactivity to food cues in the visual attention (TOFC) and reward (putamen) brain regions.

The feasibility objectives for this pilot trial were three-fold: to examine usage of the Mandolean®, tolerance of the imaging and blood sampling protocol, and ability to measure imaging signal in the brain regions of interest. The number of meals in which the Mandolean® was used during the intervention period was considerably less than planned at outset. Participants and their parents/carers commented that the Mandolean® was not always easy to use: there was no one particular challenge for participants and their carers; several issues were reported, including limiting the food that could be consumed (in terms of portion size, and type of suitable meals), requiring diners to eat at a table or near a source of power, and issues with the equipment. Moreover, one participant dropped out due to the additional time and effort to use the Mandolean® at meal times.

The imaging protocol was well tolerated by most participants. All participants agreed to have blood samples during the study consent/assent process. One volunteer decided not to take part as they were not prepared to have the blood samples taken, suggesting our informed consent/assent procedures were valid. However, it was extremely difficult to cannulate this group of obese adolescents. Samples were taken from 57% participants at the baseline scan (seven Mandolean® + group and six in standard care group) and only 42% at the post-intervention scan (four in each group). Finally, examination of the first-level brain maps for each participant showed that the imaging signal in the brain regions of interest could be measured. However, planned analyses of the relationship between the BOLD response and levels of glucose, ghrelin and PYY were not possible, due to the problems with cannulation as reported above. For the above reasons, this pilot study will not be scaled up to a full trial.

The main limitation of this study is the sample size. The planned sample size meant that it was not appropriate to perform statistical tests of differences between groups for the behavioural data, but confidence intervals for the effect sizes were included to allow interpretation at the population level. The sample size for some statistical comparisons was reduced further due to missing data due to problems with blood sampling, and data recording and collection issues with the equipment itself.

It should also be noted that there was minimal change in BMI (SDS) in both groups. However, as the intervention was conducted over a short period of 6 months, this result was not unexpected. A shorter, less intense intervention was chosen compared to the previous full RCT that was conducted over twelve months [6] to test the fMRI trial format rather than assess Mandolean® effects on weight loss. However, our findings suggest that Mandolean® training is more effective with additional support (a dedicated support nurse) aiding continued usage for a longer period (twelve rather than 6 months). The lack of large differences in BMI (SDS) post-intervention did allow the analysis of the neuroimaging and hormonal data to be conducted without confounding differences in BMI.

We acknowledge that we are unable to determine which component of Mandolean® training is responsible for the observed differences to standard care. There are elements of the training process that address meal portion size, rating of satiety during that meal and speed of food consumption on a daily basis. In addition, by choosing a simple food-cue reactivity paradigm for this study there are no direct behavioural correlates from this design (participants were not required to choose a portion size, eat a meal or rate their fullness during the scan itself). The advantage of this approach however, was to have an objective measure of food reactivity that is in line with a wealth of existing research with which to compare the effects of this behavioural intervention.

## Conclusion

In conclusion, this study provides preliminary evidence of a change in the neural response to food cues in young people with obesity after Mandolean® training to slow eating rate. These neural changes were associated with greater usage of the Mandolean®, suggesting that the more meals eaten using the Mandolean®, the greater the reduction in signal change was found in brain regions subserving visual attention and food reward in response to food cues. The implication of these neuroimaging findings is that this behavioural intervention leads to changes in the way in which individuals process food cues in the environment: by paying less attention to food cues and finding them less rewarding, individuals may be less motivated to find and eat those foods. Future work may include more imaging timepoints to allow investigation of the longevity of fMRI changes following such a behavioural intervention. Mandolean training was also associated with a reduction in portion size with no change in post-meal satiety, corroborating findings from the previous full trial (3). However, due to issues with the data collection and recording of both the blood samples and Mandolean data®, it was decided not to scale this small fMRI study to a full trial. Overall, this pilot trial supports targeting eating behaviour in weight-management interventions in young people [2, 3, 5], who are more susceptible to food cues, especially if overweight [41].
